# The spread of *Wolbachia* through mosquito populations

**DOI:** 10.1371/journal.pbio.2002780

**Published:** 2017-06-01

**Authors:** Francis M. Jiggins

**Affiliations:** Department of Genetics, University of Cambridge, Cambridge, United Kingdom

## Abstract

In many regions of the world, mosquito-borne viruses pose a growing threat to human health. As an alternative to traditional control measures, the bacterial symbiont *Wolbachia* has been transferred from *Drosophila* into the mosquito *Aedes aegypti*, where it can block the transmission of dengue and Zika viruses. A recent paper has reported large-scale releases of *Wolbachia*-infected *Ae*. *aegypti* in the city of Cairns, Australia. *Wolbachia*, which is maternally transmitted, invaded and spread through the populations due to a sperm–egg incompatibility called cytoplasmic incompatibility. Over a period of 2 years, a wave of *Wolbachia* infection slowly spread out from 2 release sites, demonstrating that it will be possible to deploy this strategy in large urban areas. In line with theoretical predictions, *Wolbachia* infection at a third, smaller release site collapsed due to the immigration of *Wolbachia*-free mosquitoes from surrounding areas. This remarkable field experiment has both validated theoretical models of *Wolbachia* population dynamics and demonstrated that this is a viable strategy to modify mosquito populations.

## Introduction

In 2008, 2 groups of researchers independently reported that a bacterial symbiont called *Wolbachia* made *Drosophila* resistant to RNA viruses [1,2]. This added to a growing list of symbionts that act as an ‘accessory immune system’, protecting insects against infection. However, both groups realized that the significance of their results went beyond insect immunity and potentially provided a new way to control mosquito-borne viruses [1,2]. As well as protecting against viruses, many *Wolbachia* strains can induce a sperm–egg incompatibility called cytoplasmic incompatibility, which allows them to rapidly spread through insect populations [3]. Therefore, if *Wolbachia* was introduced into mosquito populations, it might spread and render the mosquitoes unable to transmit pathogens like dengue virus.

This was a timely discovery, as arthropod-borne viruses (arboviruses) are a growing threat to human health. Dengue virus, which infects millions of people every year, has greatly increased its range in tropical and subtropical regions [4,5]. Chikungunya virus has become a major cause for concern after causing epidemics in Asia, Indian Ocean islands, Southern Europe, and the Americas [6]. Recently, a widespread epidemic of the Zika virus has been linked to fetal brain abnormalities [7]. In the absence of effective vaccines, efforts to control these pathogens have targeted mosquito populations. However, insecticide resistance is making this increasingly challenging, so *Wolbachia* provides a promising addition to traditional control measures.

Progress was rapid. Efforts have focused on the mosquito *Ae*. *aegypti*, which is the principal vector of dengue, Zika, chikungunya, and yellow fever viruses. Initially, there were plans to introduce a virulent strain of *Wolbachia* that would shorten the life of the mosquitoes [8], but the high fitness costs associated with this strain prevent it from being maintained in populations or spreading spatially [9,10]. As the *w*Mel strain of *Wolbachia* protected *Drosophila melanogaster* from viruses without substantial effects on lifespan [1,2], this was a natural candidate for introduction into *Ae*. *aegypti* [11]. By 2009, it had been reported that *w*Mel provided resistance against dengue virus in *Ae*. *aegypti*, preventing the virus disseminating to salivary glands, where it could be transmitted to humans [11]. As *Wolbachia* is very common in natural insect populations [12], it was possible to move to field trials without the controversy that accompanies the release of genetically modified insects.

These early field trials have sought to understand how *Wolbachia* establishes and spreads in natural populations of *Ae*. *aegypti*, and a recent paper has reported the results of a large field trial in the city of Cairns, Australia [13]. *Wolbachia*-infected mosquitoes were released for 14 weeks in 3 regions of the city, and then, the prevalence of *Wolbachia* infection was monitored for 2 years at a large number of traps around these release sites. This follows earlier releases in the Australian towns of Yorkeys Knob and Gordonvale [14]. However, these were small, isolated populations, while Cairns is a large, continuous population, which allows the spatial dynamics of *Wolbachia* to be studied.

The result has been a remarkable field experiment [13]. There is a rich theoretical literature on the dynamics of *Wolbachia* and cytoplasmic incompatibility within populations, and this dataset provides an exceptional opportunity to test this theory. The results demonstrate that these theoretical models are robust and can be used to guide public health programs that are deploying *Wolbachia* to prevent the transmission of vector-borne disease.

## The invasion of *Wolbachia*

**Fig 1:**
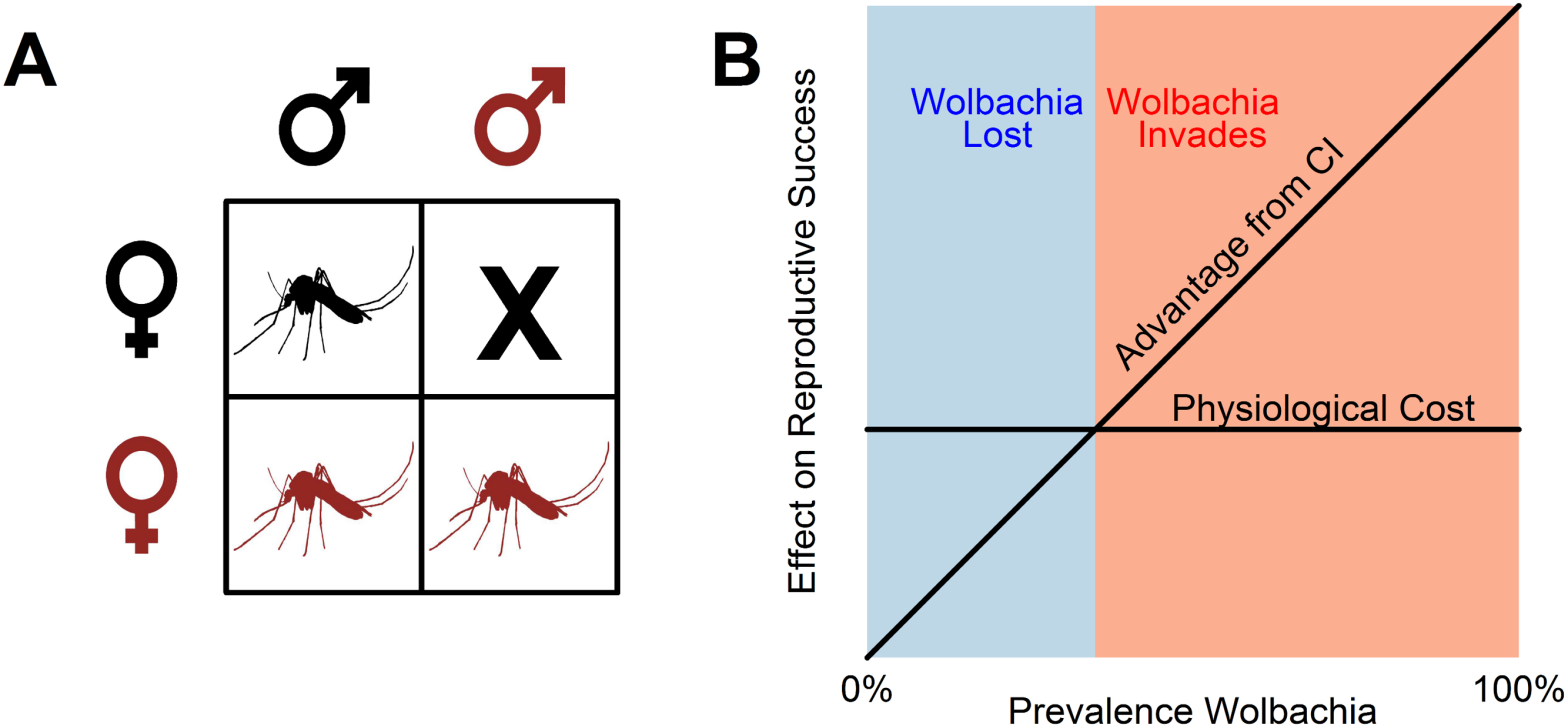
Cytoplasmic incompatibility. **(A)** When a *Wolbachia*-infected male (red) mates with an uninfected female (black), a sperm–egg incompatibility means that some or all of the embryos die. Therefore, infected females produce more offspring than uninfected females (red versus black mosquitoes). **(B)** This reproductive advantage depends on the prevalence of *Wolbachia* in the population, because when *Wolbachia* is rare, females are unlikely to mate with infected males. The *Wolbachia* strain in *Aedes aegypti* carries a physiological cost, reducing the fecundity of infected females. If this cost exceeds the advantage of cytoplasmic incompatibility, then the infection is lost from the population. This creates a threshold prevalence below which *Wolbachia* is lost and above which it invades the population. This cartoon assumes infected females transmit *Wolbachia* to all their offspring. *Image credit*: https://doi.org/10.1093/gbe/evw018.

*Wolbachia* is found within the cytoplasm of cells, and as a consequence of this, it is transmitted vertically from infected females to their offspring—males are a dead end for the symbiont. When a male is infected with a strain of *Wolbachia* that induces cytoplasmic incompatibility, its sperm are modified so that embryos die during early embryonic development (Fig 1A) [15,16]. However, in females, *Wolbachia* encodes a second factor that ‘rescues’ the embryo, allowing development to proceed normally (Fig 1A) [15,16]. This results in *Wolbachia*-infected females having a reproductive advantage, allowing the bacterium to invade and be maintained in populations.

The reproductive advantage enjoyed by *Wolbachia*-infected females is greatest when *Wolbachia* is common [17]. If the prevalence of *Wolbachia* is low, females rarely mate with *Wolbachia*-infected males, so there is little advantage to being compatible with these males. If *Wolbachia* infection is costly to the insect, then at low prevalence, these costs may outweigh the benefits of cytoplasmic incompatibility, and *Wolbachia* will be lost from the population (Fig 1B) [17]. The same occurs if infected females don’t transmit *Wolbachia* to all their offspring [17]. This results in a threshold prevalence above which *Wolbachia* will invade the population and below which it will be lost [17].

Release programs must be sufficiently large that this threshold is exceeded; otherwise, the infection will be lost when the releases stop. In *Ae*. *aegypti*, *Wolbachia* has near-perfect vertical transmission [14,18]. However, the infected females are estimated to suffer a fitness cost of about 20% [14], largely due to a reduction in their fecundity [18]. This results in a threshold prevalence of about 20%–30% [19], which must be reached by releasing infected mosquitoes, and after this point, *Wolbachia* will continue to increase in prevalence without further interventions, at least in isolated populations.

When *Wolbachia*-infected *Ae*. *aegypti* were released in Yorkeys Knob and Gordonvale in 2011 [14], the prevalence exceeded this threshold. As predicted, after releases stopped, *Wolbachia* continued to increase in frequency [14] and was then maintained in the population for over 2 years [18]. These were isolated populations, while the recent releases in Cairns are into a continuous population where mosquitoes can migrate between the release sites and surrounding *Wolbachia*-free areas [13]. Here, there was a risk that the influx of uninfected mosquitoes into the release site could push the prevalence below the threshold for invasion, leading to the loss of *Wolbachia*. However, *Wolbachia* was established and maintained well above the invasion threshold, demonstrating that the approach can succeed in a large urban area [13].

## Spatial spread of *Wolbachia*

The scale and cost of field releases will depend on whether *Wolbachia* spreads from release sites to other areas. This will occur when *Wolbachia*-infected mosquitoes disperse from the release site into neighbouring *Wolbachia*-free populations, pushing the prevalence above the invasion threshold and leading to *Wolbachia* spreading outwards in an advancing wave [20]. This was the case in the 2 largest of the 3 release sites in Cairns, where the area of the infected mosquitoes nearly doubled in 2 years [13].

The wave-like spread of *Wolbachia* out from the release site can be described by the width of the wave and the rate of its advance. Schmidt et al. [13] take a variety of approaches to estimate these parameters, ranging from simply calculating how the area occupied by *Wolbachia* changes through time to more sophisticated likelihood models that incorporated heterogeneities in the data. These different approaches yielded largely consistent estimates. After an initial phase of establishment, *Wolbachia* gradually spread out from the release sites at a constant rate of roughly 100–200 m per year, with a wave width of approximately 300–500 m [13].

These field estimates can be compared to theoretical predictions of how fast *Wolbachia* is expected to spread. The rate at which the wave of *Wolbachia* infection advances can be approximated by a simple function based on the distance mosquitoes disperse every generation and the threshold prevalence that *Wolbachia* must reach in order to invade a population [13]. The invasion threshold has been estimated previously, while the dispersal of mosquitoes between generations can be estimated from the width of the wave—the farther the mosquitoes fly, the wider the wave. It is not possible to exactly reconcile theory and data, as spread in the field was measured per day, while the theoretical rate is measured per generation. Nonetheless, with plausible generation times, the rate of spread in the field is a remarkably good match to theoretical expectations [13].

The slow spread of the *Wolbachia* through *Ae*. *aegypti* populations contrasts with the rapid spread of the *w*Ri strain of *Wolbachia* in *Drosophila simulans*. Following a natural introduction into California, *Wolbachia* swept across the state at 100 km/year, and this rapid spread was replicated 20 years later when wRi arrived in Australian populations [21]. This is nearly 3 orders of magnitude faster than has occurred in mosquito populations in Cairns [22]. This discrepancy cannot be accounted for by *Drosophila* dispersing further than *Ae*. *aegypti*, and instead, it is likely that the *w*Ri in *D*. *simulans* carried little cost or was even beneficial, so *Wolbachia* could invade from a very low prevalence [19,21]. The slow spread of *Wolbachia* in *Ae*. *aegypti* populations will make its large-scale deployment more costly and logistically challenging. One solution would be to find new *Wolbachia* strains that have a lower cost but still confer strong antiviral protection. This may be challenging, as the antiviral effects of *Wolbachia* rely on high densities of *Wolbachia* in insect tissues, and these tend to be costly [23]. As an alternative to *Wolbachia*, gene-drive systems could be used to modify mosquito populations to prevent disease transmission [24]. As these elements can typically invade populations from a low frequency, their spatial spread may be more rapid. However, such strategies are far more controversial than releasing *Wolbachia* [24].

## The effect of variation in the environment on the spread of *Wolbachia*

The rate at which *Wolbachia* spreads depends on the distance mosquitoes disperse and the cost of *Wolbachia* infection, and these are likely to be affected by environmental conditions. There was a marked difference in rate of *Wolbachia* spread out from the 2 larger release sites in Cairns, with the wave of infection advancing 186 m/year at Edge Hill compared to 110 m/year at Parramatta Park [13]. It seems most likely that this reflects differences in dispersal distances, perhaps because the habitat at Parramatta Park is better, resulting in less dispersal [13]. These differences may be far greater between sites where the climate and environment are more different. As releases are underway in several locations across the tropics, it should soon become apparent whether the results from Cairns can be generalised to other regions where arboviruses are more prevalent.

Areas of low mosquito density or barriers to dispersal can slow or halt the spread of *Wolbachia* infection through a population [20]. When an advancing wave of *Wolbachia* meets a barrier to dispersal, such as a road, this can prevent the threshold prevalence for invasion being reached in the uninfected population on the other side of this barrier [20]. Similarly, the wave of *Wolbachia* may become ‘stuck’ in a region of low population density, because the small number of migrants leaving such an area will prevent the threshold prevalence for invasion being reached in adjacent uninfected populations [20].

The rate at which *Wolbachia* spread out in different directions from the release sites in Cairns was very variable. There was even a substantial area of the largest release site where the prevalence declined towards the end of the study. Roads are known to be a barrier to *Ae*. *aegypti* dispersal, and when *Wolbachia*-infected mosquitoes were released at Gordonvale, the infection did not spread across a major highway [19]. Similarly, it failed to cross major roads in Cairns [13]. However, most of the heterogeneity remains unexplained [13]. It seems likely that unknown complexities in processes like mosquito dispersal and habitat quality will make the spread of *Wolbachia* very heterogeneous.

An important remaining question is when the expansion of *Wolbachia* from release sites will ultimately halt. Will a single release ultimately infect just the local neighbourhood, whole cities, or even spread to neighbouring settlements? Will the infection eventually jump barriers like major roads? The extent to which this matters will in turn depend on the local epidemiology of dengue viruses and whether patches of *Wolbachia*-free mosquitoes have a substantial effect on the burden of disease in the human population. These questions will have implications for the cost and design of release strategies.

## Collapse of small patches

As a patch of *Wolbachia*-infected mosquitoes gets smaller, the proportion of individuals within that patch that are immigrants from surrounding *Wolbachia*-free populations gets larger. This can result in a swamping effect, wherein the influx of uninfected mosquitoes pushes the prevalence of *Wolbachia* below the invasion threshold, resulting in the collapse of the infection. This has led to a theoretical prediction that there is a minimum area over which *Wolbachia* must be released to establish and spread [19,20]. The size of this area depends on the distance that mosquitoes disperse each generation and the invasion threshold [19,20].

The releases in Cairns were made in 3 patches of varying size, and the smallest of these was just below the theoretical minimum release area [13]. As predicted, this release showed markedly different dynamics to the 2 larger release sites. Rather than *Wolbachia* spreading outwards in an advancing wave, the area infected by *Wolbachia* roughly halved within about a year [13]. The collapse was slow, as is expected, as the area was only just below the minimum for establishment. During the study, *Wolbachia* remained above the invasion threshold, but if the collapse continues, then it is expected that the prevalence will fall below this threshold and *Wolbachia* will be lost. As this result is based on a single release, it will be important to replicate these results over more sites.
